# Antibiotic Use and Resistance Knowledge: Awareness Among the General Public in Jazan, Saudi Arabia

**DOI:** 10.7759/cureus.20369

**Published:** 2021-12-12

**Authors:** Abdulaziz Jali, Alshomokh Hakami, Najwa Dahas, Mashael Mahnashi, Afnan Siddiq, Halimah Alsomaili, Abdulaziz H Alhazmi

**Affiliations:** 1 Pharmacy, Jazan University, Jazan, SAU; 2 Medicine and Surgery, Jazan University, Jazan, SAU; 3 Microbiology, Jazan University, Jazan, SAU

**Keywords:** misuse, saudi arabia, jazan, resistance, antibiotics

## Abstract

Background and objective: Antibiotics (Abs) are a class of medication generally prescribed for bacterial infections. Abs misuse, caused by either medication non-compliance or self-medication, may lead to Abs resistance, a problem that is trending around the globe. In 2018, dispensing Abs without a prescription became prohibited in Saudi Arabia. This study aimed to evaluate knowledge and attitude toward Abs use and resistance in Jazan Province.

Materials and methods: A cross-sectional study was conducted in the population of Jazan, using a self-administered electronic questionnaire to assess the knowledge and attitude toward Abs use and resistance and its related factors.

Results: According to our findings, 543 participants responded and most of the participants use Abs with a prescription (n = 280; 75%). About 40% identified correctly that Abs can treat only bacteria, not viruses, and 44% denied that Abs can treat all coughs and common cases. Then, regarding the knowledge about resistance, we found that those who had heard about Abs resistance represented more than half of the participants (56%), and had better knowledge about Abs use. Further, more than half of those who have heard about Abs resistance correctly defined the antibiotic susceptibility test, and about two-thirds were able to answer the related questions about Abs resistance.

Conclusion: To conclude, a positive correlation was found between knowledge about Abs resistance and Abs use. Thus, national Abs regulations and policies with continuous education and awareness must be continued to ensure a better understanding of Abs use.

## Introduction

Antibiotics (Abs) are important medications that are usually indicated in cases of bacterial infection, either as prophylaxis or treatment [1]. Generally, Abs misuse is caused by non-compliance or self-medication [2]. Multidrug-resistant (MDR) organisms have been a challenge worldwide since Abs appeared and occurred through malpractice associated with Abs use [3]. In addition, approximately 700,000 people die annually due to antimicrobial resistance (AMR), which is defined as the survival of bacteria despite the presence of optimal concentration of the drug of antimicrobial agents [4]. Moreover, globally, Abs resistance increases the expenses of healthcare and requires a tremendous amount of work, factors that can affect the overall productivity of the healthcare system [5]. One of the contributing factors toward the development of Abs resistance is misuse or overuse of Abs. In an international multicenter study conducted by the WHO in 2015 to assess people’s knowledge and practices regarding Abs, the authors found that 25% of the respondents said that it is acceptable to consume Abs given by a friend or a family member, 43% believed that it is acceptable to buy the same Abs that was prescribed previously by clinicians, and 32% of respondents said that they would stop taking the Abs immediately once they feel better [6]. Thus, multiple factors link the practice of Abs misuse to the lack of compliance and accessibility to the over-the-counter Abs due to the rules and regulations that reduce such behavior [2].

Like many other countries around the globe, Abs resistance is considered a great challenge in Saudi Arabia. For example, the incidence of methicillin-resistant *Staphylococcus aureus* (MRSA) was found to be increased from 2% to 57% during the Hajj season [7]. Further, the antimicrobial stewardship programs (ASPs) are not well implemented. In a recent study, only 26% of the hospitals in Saudi Arabia reported the implementation of ASPs, and this is due to a lack the knowledge, technological and staff resources [8]. Thus, efforts from health officials and multiple national sectors are increasingly required to successfully control the AMR in the country [9]. One of these efforts that has been recently applied is the prohibition of dispensing of Abs without a prescription [10], a decision that has improved the practice toward Abs use. In one study conducted in Riyadh in 2017, the authors found that there is a variation in Abs resistance knowledge among Saudi population. Approximately 67% did not know what Abs resistance means, 67% denied that Abs can damage children’s teeth, and 65% were unaware that Abs overuse might lead to morbidity and mortality. Furthermore, they found that almost 51% of people used Abs without a prescription and 37.5% obtained Abs from pharmacists without prescriptions [11]. Likewise, another study was conducted in Makkah in 2019 to assess the Knowledge, Attitude and Practices (KAP) regarding the use of Abs for upper respiratory tract infections (URTIs). The survey showed that 70% of parents believed that physicians were a source of information about Abs use; however, only 8% agreed that most URTIs are caused by viruses, and approximately 53% of parents expected pediatricians to prescribe Abs for the general symptoms of upper respiratory tract infections regardless of the etiology [12].

Despite many studies about Abs misuse and resistance knowledge from some regions around the globe, including Saudi Arabia, there are a limited number of articles discussing the knowledge of Abs misuse and resistance among the population of Jazan. Therefore, we aimed to evaluate the knowledge of Abs misuse and Abs resistance in the Jazan Province in the southwestern region of Saudi Arabia.

## Materials and methods

Study design and setting

A cross-sectional, observational study was carried out in the population of Jazan Province between 27 August and 5 September 2021. The public was invited to participate in an online questionnaire that was generated after an extensive review of the literature and related studies [2,6]. The questionnaire was designed electronically as a Google Form and the invitation was distributed via social media, based on the investigators’ networks.

Inclusion and exclusion criteria

Those who lived in Jazan City aged 18 years or older who were willing to participate voluntarily in this study were included. We excluded those who lived outside Jazan and those who refused to participate.

Sample size

The Raosoft sample size calculator (Raosoft Inc., Seattle, WA, USA) was used to calculate the sample size for this study. The population of Jazan Province is around two million inhabitants. The sample size was calculated based on a 5% margin of error, a 50% response rate, and a 95% confidence interval. Consequently, a sample size of 323 responses was determined to be sufficient for this study. However, to reduce sampling bias in our method as an online study, we increased the sample size to include approximately 500 participants. Prior to distribution of the survey, a pilot sample (n = 30) was used to evaluate the clarity and the wording of the questionnaire items. Of note, data from this pilot sample were excluded from the study analysis.

Survey instrument

The questionnaire was divided into four sections. The first and second sections asked for demographic details (such as age, gender, education level, monthly income in Saudi Riyals, and profession) and some questions about Abs use in the last 12 months. The second section assessed the knowledge about Abs. The third section assessed the knowledge of participants regarding Abs resistance. We used validated questionnaires to assess Abs resistance and the used questionnaire is in the appendix [2,6].

Statistical analysis

Data were collected and checked regularly by the researchers. The Statistical Package for Social Sciences (SPSS) version 23 (IBM Corp., Armonk, NY, USA) software program was used for data analysis. Frequency distributions were obtained, and descriptive statistics were calculated. Another level of data analysis (chi-square test) was used to test some associations. A p-value of less than 0.05 was considered significant. Multivariate logistic regression models were applied to variables that might be related to the knowledge about Abs resistance from which we obtained adjusted odds ratio (aOR), 95% confidence intervals (CI).

Ethical approval

Ethical approval for conducting this study was obtained from the ethical approval committee at Security Forces Hospital (reference number, H-01-R-069; date, 17 April 2019). Consent was obtained from all participants prior to participation in the study.

## Results

The study included 543 participants; most of them (99%) agreed to participate in this study and the majority were females (n = 385; 71%). The vast majority of the participants (76%) held a Bachelor’s degree, and approximately 45% of the participants were students. Approximately 70% of the participants had used Abs in the last 12 months, and the majority of them received professional advice on how to use Abs and obtained them from a pharmacy. The additional sociodemographic factors are listed in Table 1.

**Table 1:** General characteristics of the participants in our study. SD, standard deviation; HCW, healthcare workers; SAR, Saudi Riyal; Abs, antibiotics.

Variable	Participants, n = 543
Age, years (median; SD)	25; 7
Male, n (%)	158 (29%)
Profession	
HCW	47 (9%)
Non-HCW	109 (20%)
Student	246 (45%)
Freelancer	18 (3%)
Housewife	55 (10%)
Unemployed	60 (11%)
Retired	8 (2%)
Residence, n (%)	
City	286 (53%)
Village	257 (47%)
Marital status, n (%)	
Single	287 (53%)
Married	241 (44%)
Divorced	12 (2%)
Widowed	3 (1%)
Monthly income (SAR), n (%)	
Less than 3000	287 (53%)
3000 to 5000	54 (10%)
5000 to 10,000	80 (15%)
10,000 to 20,000	105 (19%)
More than 20,000	17 (3%)
Education level, n (%)	
Uneducated	5 (1%)
High school	108 (20%)
Bachelor’s degree	412 (76%)
Postgraduate degree	18 (3%)
Used Abs in the last 12 months, n (%)	
No	169 (31%)
Yes	374 (69%)
Last month	154 (28%)
Last 6 months	125 (23%)
Last 12 months	95 (18%)
Of these, used Abs with a prescription	
Yes	280 (75%)
No	94 (25%)
Received advice from a doctor or pharmacist on how to use Abs	
Yes	307 (82%)
No	18 (67%)
Place from which Abs were obtained	
Pharmacy	299 (82%)
Family or friends	30 (8%)
Internet	2 (1%)
Previous prescription	35 (10%)

Among those who used Abs in the last 12 months, most of the participants used Abs with prescriptions (n = 280; 75%). Next, we compared those who used Abs with a prescription and those who obtained Abs without a prescription. We found that gender and profession significantly affected using Abs without a prescription. In addition, we found that obtaining Abs with a prescription was significantly associated with receiving advice on how to use Abs by a doctor or pharmacist, and with obtaining Abs from a pharmacy (Table 2).

**Table 2:** Univariate analysis of the participants who used Abs without a prescription compared to those who used it with a prescription SD, standard deviation; HCW, healthcare workers; SAR, Saudi Riyal; Rx, prescription. # The alpha criterion for the p-value was set to 0.05. * Significant in univariate analysis.

Variable	Participants Who Used Abs without Rx n = 94 (25%)	Participants Who Used Abs with Rx n = 280 (75%)	p-Value ^#^
Age, years (median; SD)	26;9	24;12	0.139
Male, n (%)	47 (50%)	70 (25%)	0.0001 *
Profession			0.038 *
HCW	8 (9%)	25 (9%)	
Non-HCW	29 (31%)	62 (22%)	
Student	44 (47%)	106 (38%)	
Freelancer	2 (2%)	9 (3%)	
Housewife	2 (2%)	33 (12%)	
Unemployed	7 (7%)	39 (14%)	
Retired	2 (2%)	6 (2%)	
Residence, n (%)			0.153
City	40 (43%)	143 (51%)	
Village	54 (57%)	137 (49%)	
Marital status, n (%)			0.462
Single	51 (54%)	130 (46%)	
Married	40 (43%)	141 (50%)	
Divorced	3 (3%)	7 (3%)	
Widowed	0	2 (1%)	
Monthly income (SAR), n (%)			0.428
Less than 3000	43 (46%)	143 (51%)	
3000 to 5000	10 (11%)	29 (10%)	
5000 to 10,000	14 (15%)	48 (17%)	
10,000 to 20,000	26 (27%)	53 (19%)	
More than 20,000	1 (1%)	7 (3%)	
Education level, n (%)			0.283
Uneducated	0	3 (1%)	
High school	15 (16%)	52 (19%)	
Bachelor’s degree	78 (83%)	213 (76%)	
Postgraduate degree	1 (1%)	12 (4%)	
Received advice from a doctor or pharmacist on how to use Abs			0.0001 *
No	46 (49%)	21 (8%)	
Yes	48 (51%)	259 (92%)	
Place where Abs were obtained			0.0001 *
Pharmacy	46 (49%)	261 (93%)	
Family or friends	20 (21%)	10 (3%)	
Internet	0 (0%)	2 (1%)	
Previous prescription	28 (30%)	7 (3%)	

We assessed the knowledge about Abs use. We found that approximately 43% said that Abs use could be stopped when they feel better, and more than one-third (38%) of the participants answered that Abs were used to treat bacteria. One-third of the participants believed that Abs can treat common colds and coughs (Table 3). Moreover, those participants who had heard about bacterial resistance to Abs made up more than half of the respondents (n = 302; 56%) compared to those who denied knowing about Abs resistance (44%).

**Table 3:** Knowledge toward Abs use Abs: antibiotic.

Questions	Participants, n = 543
Stop the use of Abs when, n (%)	
I feel better	232 (43%)
Doses completed as prescribed	258 (47%)
I don’t know	53 (10%)
Abs can treat, n (%)	
Bacteria	209 (38%)
Viruses	77 (14%)
Both	187 (34%)
I don’t know	119 (22%)
Can Abs treat all cough and cold cases?, n (%)	
Yes	160 (30%)
No	240 (44%)
I don’t know	143 (26%)
Have you ever heard of bacterial resistance to Abs?, n (%)	
Yes	302 (56%)
No	241 (44%)

We then aimed to measure the factors and practices associated with knowledge about Abs resistance (Table 4). Using univariate analysis, we found that profession and education were significantly associated with knowledge of Abs resistance. Furthermore, those who had heard about Abs resistance correctly answered questions about Abs use. For example, they said that the prescribed Abs should be stopped when the course was completed and they did not believe that Abs can treat common cold cases. Using multivariate analysis, stopping the prescribed Abs when the course was completed and believing that Abs can treat common cold cases were significantly associated with the knowledge about Abs resistance (Table 4). The same findings, stopping the prescribed Abs when the course was completed and believing that Abs can treat common cold cases were significantly associated with the knowledge about Abs resistance, were also significant in multivariate analysis (Table 4).

**Table 4:** Univariate and multivariate analysis of the participants who had heard about bacterial resistance to Abs versus those who denied knowing about Abs resistance. SD, standard deviation; Abs, antibiotics; SAR, Saudi Riyals. # The alpha criterion for the p-value was set to 0.05. * Significant in univariate analysis. ** Significant in Multivariate analysis. & Chi-squared test and t-test were used for univariate analysis. && Multiple logistic regression was used for multivariate analysis using the variable significantly associated with the knowledge about Abs resistance.

	Univariate analysis^&^			Multivariate analysis^&&^	
Variable	Did Not Heard about Abs Resistance, n = 241 (44%)	Heard about Abs Resistance, n = 302 (56%)	p-Value ^#^	aOR	95% CI
Age, years (median; SD)	29; 11	29; 12	0.955	-	-
Male, n (%)	69 (23%)	89 (37%)	0.85	-	-
Profession, n (%)			0.014 *	-	-
HCW	12 (5%)	35 (12%)			
Non-HCW	49 (20%)	60 (20%)			
Student	101 (42%)	145 (48%)			
Freelancer	10 (4%)	8 (3%)			
Housewife	32 (13%)	23 (8%)			
Unemployed	32 (13%)	28 (9%)			
Retired	5 (2%)	3 (1%)			
Residence, n (%)			0.227	-	-
City	107 (44%)	150 (50%)			
Village	137 (56%)	152 (50%)			
Marital status, n (%)			0.661	-	-
Single	121 (50%)	166 (55%)			
Married	114 (47%)	127 (42%)			
Divorced	5 (2%)	7 (2%)			
Widowed	1 (1%)	2 (1%)			
Monthly income (SAR), n (%)			0.169	-	-
Less than 3000	127 (53%)	160 (53%)			
3000 to 5000	27 (11%)	27 (9%)			
5000 to 10,000	42 (17%)	38 (13%)			
10,000 to 20,000	37 (16%)	68 (23%)			
More than 20,000	8 (3%)	9 (2%)			
Education level, n (%)			0.002 *	-	-
Uneducated	5 (2%)	0			
High school	59 (25%)	49 (16%)			
Bachelor’s degree	173 (72%)	239 (79%)			
Postgraduate degree	4 (1%)	14 (5%)			
Used Abs in the last 12 months, n (%)			0.514	-	-
No	71 (29%)	98 (32%)			
Yes	170 (71%)	204 (68%)			
Stop use of Abs when, n (%)			0.0001 *		
I feel better	118 (49%)	114 (38%)		0.415**	0.200-0.862
Doses completed as prescribed	86 (36%)	172 (57%)		0.247**	0.118-0.517
I don’t know	37 (15%)	16 (5%)		-	-
Can Abs treat all coughs and cold cases?, n (%)			0.0001 *		
No	65 (27%)	175 (58%)		0.207**	0.127-0.337
Yes	77 (32%)	83 (27%)		0.474**	0.286-0.785
I don’t know	99 (41%)	44 (15%)		-	-

Those participants who had heard about bacterial resistance to Abs (n = 302) were guided to further questions to assess their knowledge about Abs resistance. According to our findings, 50% believed that the Abs susceptibility test is used to ensure that Abs are sensitive against a specific type of bacteria, whereas 15% believed it was used to ensure the safety profile of Abs. Furthermore, 93% thought that Abs resistance occurs when bacteria develop mechanisms that protect them from the effects of Abs, 85% believed that many infections are becoming increasingly resistant to Abs, and 84% believed that Abs-resistant infections are not easily treated. Furthermore, approximately two-thirds (72%) stated that Abs resistance is a problem that exists in many countries of the world, including Saudi Arabia. In addition, approximately 63% believed that Abs-resistant bacteria can be transmitted from person to person (Table 5).

**Table 5:** Knowledge about Abs resistance. Abs: antibiotics

Questions	Participants, n = 302
The Abs susceptibility test is, n (%)	
To ensure the safety profile of Abs	45 (15%)
To ensure that Abs are sensitive against a specific type of bacterium	152 (50%)
To find out the effectiveness of Abs against specific cells	44 (15%)
I don’t know	61 (20%)
Abs resistance occurs when bacteria develop mechanisms that protect them from the effects of Abs, n (%)	
TRUE	281 (93%)
FALSE	21 (7%)
Many infections are becoming increasingly resistant to Abs, n (%)	
TRUE	258 (85%)
FALSE	44 (15%)
If bacteria are resistant to Abs, it can be difficult to treat the infection they cause, n (%)	
TRUE	255 (84%)
FALSE	47 (16%)
Abs resistance is a problem that can affect me or my family, n (%)	
TRUE	248 (82%)
FALSE	54 (18%)
Abs resistance is a problem that exists in many countries of the world, but we do not have it in Saudi Arabia, n (%)	
TRUE	83 (28%)
FALSE	219 (72%)
Abs-resistant bacteria can spread from person to person, n (%)	
TRUE	190 (63%)
FALSE	112 (37%)

## Discussion

Multiple studies have been conducted at national and international levels to evaluate the public knowledge about Abs misuse and resistance and to illustrate its possible dangerous consequences. In this study, we aimed to fill a gap in knowledge, since there are limited data that reflect the public knowledge regarding Abs use and resistance in Jazan Province. In Saudi Arabia, dispensing Abs without a prescription has not been allowed since May 2018 [10]. Furthermore, before this law was applied, approximately 70% of community pharmacists admitted that giving Abs without a prescription was common. However, after the law had been passed, studies found that only 13% prescribed Abs without a prescription [13]. In our study, 75% of the participants used Abs with a prescription and most of them obtained Abs from a pharmacist and received professional advice on how to use them. This finding is significantly different from that of a previous study that was conducted in Riyadh in 2017, i.e., prior to the restriction of Abs use, in which almost 51% used Abs without a prescription, while approximately 38% of the participants in the study obtained Abs directly from pharmacists without a physician’s prescription, and, notably, only 8% used Abs with a prescription [11]. Therefore, it seems that the restriction of Abs prescriptions has been impactful [13], as we found that neither the level of education nor the place of residence had a direct impact on using Abs without a prescription (Table 2). Moreover, the main source for Abs in our study was a pharmacy (82%), and only a few of the participants (8%) obtained Abs from family or friends. In Jordan, a country that has no such obligations regarding Abs use, a study that was recently conducted in 2021 found that although pharmacies represented the source of Abs for approximately half of the participants, approximately 24% obtained Abs from a family member or a friend [4]. Thus, national regulations regarding Abs use must be continued and effectively monitored in Saudi Arabia, a country that has religious seasons and great challenges of welcoming millions of people periodically [8].

In our study, it was obvious that the participants were confused about the use of Abs as a treatment for either bacteria or viruses, since approximately 38% believed that they were used for bacterial infections, and 34% believed they could be used for both microbes. These findings are largely consistent with another study that was conducted in Riyadh in 2017, in which 38% believed that Abs were used for bacterial infections, 24% said that Abs worked on viruses, and 31% believed that Abs could treat the common cold [11]. This misconception in our region may be due to ineffective communication during counseling, as doctors sometimes use general terms when prescribing Abs and infections, instead of directly specifying bacteria as the causative organisms [14]. In addition, many participants did not recognize the differences between bacteria and viruses, and they believed that Abs are adequate for treating all infections, such practice can lead to bacterial selective pressure and Abs resistance [14,15]. Thus, doctors should prioritize effective communication with the patients about Abs use, using simple and clear terminology [14]. Moreover, national awareness programs about Abs use and resistance must be conducted and directed at the general population by the health officials to assure better attitude toward Abs use [8], as its demonstrated in our study (Table 4), in which we found that, using univariate and multivariate analysis, participants who had heard of Abs resistance knowing that the prescribed Abs should be stopped when the course is completed (57%; p = 0.0001) and believing that Abs cannot treat all cough and common cold cases (58%; p = 0.0001). This observation, i.e., the positive association between the knowledge about Abs resistance, Abs usage, is in line with prior studies. For example, a positive correlation was found between knowledge and attitudes in a Malaysian study [16]. Moreover, in a Korean study, the authors found that proper attitudes toward Abs use were a predictor of an adequate understanding of Abs use, in which participants with sufficient knowledge were more likely to express appropriate attitudes [17].

In our study, the participants who had heard about Abs resistance (n = 302) were subjected to further knowledge assessment on Abs resistance, and the results clearly showed that most of the respondents correctly believed that bacteria develop mechanisms to protect them from the effects of Abs (93%), and the majority of them indicated that many infections are becoming increasingly resistant to Abs (85%) (Table 5). They also correctly claimed that if bacteria are resistant to Abs, it can be difficult to treat the infections they cause (84%). However, in the multicenter study conducted by the WHO about whether the participants had or had not heard about Abs resistance, the authors found that 72% of respondents correctly identified that many infections are becoming increasingly resistant to treatment by Abs, and 70% thought that when bacteria are resistant to Abs, they can be very difficult or impossible to treat [6]. In addition, Abs resistance is a global problem that is trending in most countries. To illustrate this information and its association with knowing about Abs resistance, a question was directed at respondents who knew or had heard about Abs resistance whether Abs resistance was not a current problem in Saudi Arabia. Most of the respondents (72%) correctly believed that this was a false statement (i.e., they believed Abs resistance was a problem in Saudi Arabia). In contrast, in the Jordanian study where participants had either heard or not heard about Abs resistance, 53% believed that Abs resistance was a problem in other countries but not in Jordan [4]. Similarly, in Kuwait, nearly more than half of the participants disagreed that Abs resistance was a worldwide issue [18]. These findings indicate the real need to continue national and international education and awareness programs regarding Abs resistance, in which social media could be exploited to enhance the knowledge about Abs use and the dangers of Abs resistance, which could become a global pandemic [19]. Moreover, a more effective educational program could be enhanced by the participation of pharmacists and physicians, an action that would play a significant role in improving public perceptions and behavior regarding Abs misuse and resistance [5].

This study is one of the few studies targeting the general public in Jazan to assess the current knowledge regarding Abs misuse in this province and is one of the few studies in Saudi Arabia to discuss Abs resistance knowledge. We believe that this may enhance the perceptions of Abs use and pave the way for targeted programs of health promotion and Abs awareness campaigns. Furthermore, our sample size (n = 543) represented a good number that is likely to reflect the province’s population [20]. However, there are many limitations. This study was based on an online survey through social media, which may have led to a non-response bias due to the barrier of Internet accessibility in some areas and a certain type of population. In addition, the over-representation of females, students, and those with a Bachelor’s degree, which could have had an impact on the target population reflected in the study, may have been due to the data collectors mostly being female students who used their networks to spread the survey. Further, it would beneficial if the survey succeeded to include clear questions about attitudes and practices toward Abs use. On the contrary, this study recommends increasing public awareness campaigns regarding Abs use and resistance. In addition, further studies of the Saudi population at a national level are required to measure the knowledge, awareness, and attitudes toward the use of Abs and Abs resistance.

## Conclusions

In conclusion, it seems that better awareness of Abs resistance will have a direct reflection on the knowledge and attitude toward using Abs. Most of the participants in our study used Abs with a prescription and pharmacy represents the main source of Abs without a prescription, a practice that was greatly enhanced when health officials in Saudi Arabia succeeded in applying a law to prohibit dispensing Abs without a prescription. Since there are limited data published in Jazan or other provinces in Saudi Arabia, we emphasize that further studies on Abs misuse should be conducted. It is important to encourage the health authorities to implement further ASP educational interventions and target public knowledge and awareness by enhancing health promotion campaigns and delivering simple messages regarding best practices regarding Abs use.

## Appendices

I agree to participate in the survey? *

Yes

No

age *

.......

gender *

Male

Female

Occupation? *

health worker

unhealthy employee

student

free work

House wife

Job Seekers

retired

housing *

city

village

Status? *

single/single

married

divorced

widow

Monthly Income in Riyals *

less than 3000

From 3 thousand to 5 thousand

From 5 thousand to 10 thousand

From 10 thousand to 20 thousand

More than 20 thousand

education level? *

uneducated

Intermediate or secondary

university

Postgraduate

Have you used an antibiotic in the last 12 months? *

I have never used an antibiotic

in the last month

6 months ago

12 months ago

Questions about information about antibiotics

That time you used an antibiotic, did you take it on prescription? *

Yes

No

The time you used an antibiotic, did you get advice from a doctor, nurse or pharmacist about how to take it? *

Yes

No

The time you used the antibiotic, where did you get the antibiotic from? *

pharmacy

From a friend or family member

Internet

I have leftovers from previous packages

General questions about the use of antibiotics

Should I stop taking the antibiotic when? *

I felt better and the symptoms are gone

All antibody doses have been completed as prescribed

I do not know

Antibiotics treat the diseases they cause (you can choose more than one option) *

bacteria

Viruses

Bacteria and viruses together

I do not know

Do antibiotics treat all cases of coughs and colds? *

Yes

No

I do not know

Do you read the leaflet that comes with the antibiotic package? *

Yes

No

Have you ever "intentionally" changed the type or dose of your antibiotic during treatment? (without professional advice) *

Yes

No

Have you ever heard of bacterial resistance to antibiotics? *

Yes

No

General questions regarding antibiotic resistance

What is the sensitivity test of bacteria to antibiotics? *

To ensure the safety of the antibiotic and its toxicity

To ensure that the antibiotic is effective against a specific type of bacteria

To know the size of the effect of an antibiotic on a specific type of body cell

I do not know

Antibiotic resistance occurs when bacteria develop mechanisms that protect them from the effects of antibiotics *

Right

Wrong

Many infections are becoming increasingly resistant to antibiotic treatment *

Right

Wrong

If bacteria are resistant to antibiotics, it can be difficult to treat the infection they cause *

Right

Wrong

Antibiotic resistance is a problem that can affect me or my family *

Right

Wrong

Antibiotic resistance is a problem that exists in many countries of the world, but we do not have it in Saudi Arabia *

Right

Wrong

Antibiotic-resistant bacteria can spread from person to person *

Right

Wrong
